# The m6 RNA methylation regulator KIAA1429 is associated with autophagy‐mediated drug resistance in lung cancer

**DOI:** 10.1096/fba.2023-00083

**Published:** 2024-03-15

**Authors:** Bo Ma, Lei Xiu, Lili Ding

**Affiliations:** ^1^ Department of General Thoracic Surgery General Hospital of Ningxia Medical University Yinchuan China; ^2^ Department of Thoracic and Cardiac Surgery General Hospital of Ningxia Medical University Yinchuan China; ^3^ Department of Obstetrics and Gynecology Examination General Hospital of Ningxia Medical University Yinchuan China

**Keywords:** autophagy, drug resistance, KIAA1429, non‐small‐cell lung cancer, WTAP

## Abstract

N6‐methyladenosine (m6A) modification plays a crucial role in cancer progression. However, the role of m6A modification‐mediated autophagy underlying non‐small cell lung cancer (NSCLC) gefitinib resistance remains unknown. Here, we discovered that m6A methyltransferase KIAA1429 was highly expressed in NSCLC gefitinib‐resistant cells (PC9‐GR) as well as tissues, and KIAA1429 high expression was associated with poor survival. In addition, silent KIAA1429 repressed gefitinib resistance in NSCLC and reduced tumor growth in vivo. Mechanistically, KIAA1429 stabilized WTAP, a significant player in autophagy, by binding to the 3′ untranslated regions (3′‐UTR) of WTAP. In a word, our findings indicated that KIAA1429 could elevate NSCLC gefitinib resistance, which may provide a promising targeted therapy for NSCLC patients.

## Graphical abstract

KIAA1429 promotes autophagy in NSCLC by stabilizing WTAP mRNA in an m6A independent manner.

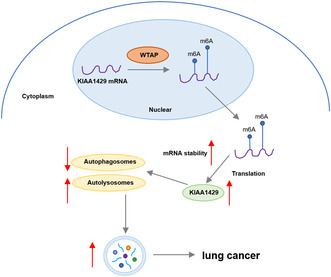

## 1 INTRODUCTION

Non‐small‐cell lung cancer (NSCLC) is the primary subtype of lung cancer^1^, ^2^ and the most common malignancy with the highest number of deaths in the world.^3^, ^4^ The primary reason is that chemotherapy and targeted therapy have lost efficacy in clinical treatment.^5^ Epidermal growth factor receptor tyrosine (EGFR) kinase inhibitors are early‐stage targeted therapies for advanced NSCLC patients with EGFR mutations, including gefitinib together with erlotinib.^6^ One disadvantage is that gefitinib resistance in NSCLC results in therapeutic impedance.^7^ Hence, it is crucial to explore the possible mechanisms of gefitinib resistance in patients with NSCLC.

N6‐methyladenosine (m6A) belongs to a reversible process modulated by methyltransferases, demethylases, and effector proteins.^8^, ^9^, ^10^, ^11^ Emerging evidence conclusively demonstrated the potential of the m6A enzyme to modulate cancer activity and progression. For instance, METTL14 promotes migration and proliferation by modulating PERP in an m6A dependent manner.^12^ Wilms tumor 1‐associating protein (WTAP), METTL3, along with METTL14 constructed a highly conserved poly‐subunit methylase complex that mediate adenosine conversion to m6A. Nevertheless, KIAA1429, also thought to be VIRMA (vir‐like m6A methyltransferase‐associated protein), is a renowned subtype of m6A methyltransferase in human cancers and has been found to be carcinogenic in many cancers. For instance, KIAA1429 increases gastric cancer cells proliferation by modulating the stability of c‐Jun mRNA in a m6A‐independent manner.^13^ KIAA1429 inhibits ID2 by upregulating the m6A modification of ID2 mRNA in hepatocellular carcinoma, thereby promoting migration and invasion of hepatocellular carcinoma.^14^ Interestingly, KIAA1429 plays an important role in NSCLC. For instance, Huang et al. revealed that KIAA1429 promotes tumorigenesis and gefitinib resistance in lung adenocarcinoma by activating the JNK/MAPK pathway in an m6A‐dependent manner.^15^ Chen et al. showed that silencing of m6A methyltransferase KIAA1429 inhibits the progression of NSCLC by promoting the p53 signaling pathway and ferroptosis.^16^

Autophagy is an evolutionarily conserved intracellular degradation and metabolism process that plays important roles in maintaining cell metabolism, genome integrity, and organelles self‐renewal.^17^, ^18^ At the same time, autophagy is also a double‐edged sword in tumor cells. Inhibition of autophagy increases the susceptibility of cancer cells to anti‐cancer therapy, but excessive autophagy can lead to autophagic cell death. Therefore, autophagy may be a potential therapeutic target for the treatment of tumors. However, abnormal regulation of autophagy can cause the pathogenesis of numerous human diseases, including cancers.^19^, ^20^ It is known that WTAP, a member of the M6A methyltransferase complex, is a potential target for KIAA1429 and a significant player in autophagy.^21^, ^22^ Likewise, gefitinib can reduce lysosomal acidification, autophagosomes, and lysosomal fusion, resulting in hindering autophagy.^23^ Therefore, exploring the regulatory mechanism between autophagy and KIAA1429 may become a new therapeutic target for NSCLC.

In this study, we aim to elucidate the critical regulatory mechanisms of the expression and the underlying mechanisms KIAA1429 resistance to gefitinib in NSCLC. We found that KIAA1429 can act as an oncogene in NSCLC by stabilizing WTAP mRNA in an m6A independent manner. This highlights a functional role of KIAA1429 as a potential prognostic biomarker and therapeutic target in NSCLC.

## 2 MATERIALS AND METHODS

### 2.1 Clinical samples

The retrospective study enrolled a total of 14 patients, aged 37 to 68 years, who underwent surgical resection and were diagnosed with NSCLC. The Ethics Committee of the General Hospital of Ningxia Medical University approved this study. Written informed consent was acquired from all patients. Tumor tissue samples were obtained during surgery. The NSCLC clinicopathological features are indicated in Table 1.

**TABLE 1:** Clinicopathological feature of NSCLC patients with elevated KIAA1429 expression.

Variable		KIAA1429 expression		*p* Value
		Low (*n* = 7)	High (*n* = 7)	
Gender				
Male	9	4	5	>0.9999
Female	5	3	2	
Age (year)				
>60	10	6	4	>0.9999
<60	4	2	2	
TNM				
I–II	6	2	4	>0.9999
III–IV	8	3	5	
Differentiation				
Well/moderate	7	2	5	>0.9999
Poor	7	3	4	
Lymphatic metastasis				
Yes	9	2	7	>0.9999
No	5	4	1	

*Note*: *p* < 0.05 statistically different.

### 2.2 Cell culture

Normal human bronchial epithelial cells (NHBE) as well as NSCLC cell lines (PC9, gefitinib‐resistant PC9/GR) were obtained from Procell (Wuhan, China). Cells were cultivated in DMEM (Beyotime, Shanghai, China) containing 10% FBS (Beyotime), 1% penicillin/streptomycin, and 25 mmol/L glucose at 37°C with 5% CO_2_.

### 2.3 Cell transfection

KIAA1429 overexpression plasmid was constructed by obtaining KIAA1429 full‐length cDNA (gene ID: NM_015496), amplifying and cloning the lentiviral vector pLenti‐copGFP‐P2A‐PuroCMV‐MCS‐3Flag (GenePharma, Shanghai, China). Lentiviral vectors containing KIAA1429 shRNA were obtained from GenePharma (sh‐KIAA1429‐1: 5′‐ATTCCGAGCTAATTCAAGATTT‐3′; sh‐KIAA1429‐2: 5′‐TTAAGCGCTACCGGGATTT‐3′; sh‐KIAA1429‐3: 5′‐ACCTGGTTCCAAGCACGCTTTTT‐3′). In addition, shRNAs targeting WTAP was produced by GenePharma and transfected using Lipofectamine 2000 reagent (Thermo fisher Scientific).

### 2.4 Quantitative real‐time PCR (qRT‐PCR)

Total RNA from NSCLC cells and tissues was isolated using the miRNeasy Mini Kit (Qiagen, USA). Total RNA (1 μg) was reverse‐transcribed to cDNA using an RNA cDNA Kit (Applied Biosystems, USA) followed by PCR with SYBR Green PCR Master Mix (Applied Biosystems). The utilized primers in this study were as indicated:

KIAA1429: Forward: 5′‐AAGTGCCCCTGTTTTCGATAG‐3′; Reverse: 5′‐ACCAGACCATCAGTATTCACCT‐3′.

WTAP: Forward: 5′‐CTTCCCAAGAAGGTTCGATTGA‐3′; Reverse: 5′‐TCAGACTCTCTTAGGCCAGTTAC‐3′.

β‐Actin: Forward: 5′‐CTCCATCCTGGCCTCGCTGT‐3′; Reverse: 5′‐GCTGTCACCTTCACCGTTCC‐3′.

β‐Actin was utilized as the internal control. Gene expression was calculated using the 2^−ΔΔCt^ method.

### 2.5 Western blot analysis

Total protein was extracted from NSCLC cells using radio‐immunoprecipitation assay (RIPA) buffer (Beyotime). Proteins were electrophoresed on 10% SDS‐PAGE, and the electrophoresed proteins were transferred to a polyvinylidene fluoride (PVDF) membrane (Millipore, USA). Membranes were blocked with 5% non‐fat milk and followed by probing with primary antibodies (anti‐KIAA1429, anti‐WTAP, and anti‐β‐actin) and corresponding secondary antibody. The blots were visualized using ECL and quantified using Image Studio software.

### 2.6 Cell migration assay

PC9‐GR cells were cultured and grown in six‐well plates until they reached 80% confluence, followed by manually wounding using a 200 μL pipette tip. The monolayer was incubated at 37°C with fresh medium after washing. Wound closure was imaged and calculated.

### 2.7 Cell Counting Kit‐8 (CCK‐8) assay

The effect of silencing KIAA1429 on the gefitinib drug sensitivity was detected using CCK‐8 and the IC50 was calculated. Briefly, PC9‐GR cells (1.5 × 10^4^/well) were plated in 96‐well plates and gefitinib at the indicated concentration was added to the cells. The Cell Counting Kit‐8 (Abcam, Cambridge, USA) was implemented to test OD values at 450 nm using a microplate reader.

### 2.8 m^6^A quantification in total RNA

To analyze the relationship between m6A methylation levels and KIAA1429 expression in NSCLC cells, the m6A RNA Methylation Quantification Kit (Abcam) was carried out for m6A methylation of total RNA. Total RNA was isolated from cells using TRIzol (Beyotime). After binding 200 ng RNA to the wells for 90 min at 37°C, the samples were incubated for 60 min with capture antibody at room temperature according to the manufacturer's instructions. Then, the RNA mixture was incubated with the enhancement solution at room temperature for 30 min after the incubation with detection antibody. Once the detection signal transduction was complete, the m6A colorimetric quantification of m6A levels was performed at an absorbance of 450 nm on a microplate reader FlexStation III (Molecular Services, USA) within 2 to 15 min.^24^

### 2.9 RNA immunoprecipitation (RIP) assay

Assay was processed based on Magna RIP RNA‐Binding Protein Immunoprecipitation Kit (Sigma‐Aldrich, USA) instruction. Cell lysate was collected and treated with protein A/G beads coated with anti‐KIAA1429‐specific as well as normal IgG antibody at 4°C overnight, followed by RT‐qPCR analysis.

### 2.10 m^6^A‐RNA immunoprecipitation assay (MeRIP‐qPCR)

Briefly, total RNA was extracted from cells, then fragmented to 100‐300‐nt and followed by incubated with m6A antibody (Abcam) or anti‐IgG‐conjugated with protein A/G magnetic beads at 4°C overnight. After elution and purification, RNA was reverse‐transcribed using the SuperScript First‐Stand Synthesis system (Thermo Fisher Scientific) followed by quantitative RT‐PCR.

### 2.11 RNA stability

WTAP mRNA transcription was inhibited by the addition of Actinomycin D (2 mg/mL) or a negative control (DMSO) followed by RT‐qPCR.

### 2.12 In vivo analysis

Male BALB/c nude mice (6‐week‐old) were purchased from Beijing Vital River. The Ethics Committee of the General Hospital of Ningxia Medical University approved this experiment. PC9‐GR cells stably transfected with sh‐KIAA1429 or control were suspended in PBS (100 μL). Cells were injected into the flanks of BALB/c nude mice, and animals were kept in a germ‐free environment until tumor formed. Tumor volumes were measured every 3 days. The animals were euthanized after 4 weeks, and tumor weight was recorded.

### 2.13 FACS analysis

3 × 10^5^ cells/well were seeded in 6‐well plates and then resuspended. Equal amounts of FITC‐Annexin V and PI were added to the cells for cultivation in the dark and followed by observation using flow cytometry.

### 2.14 Transmission electron microscopy (TEM)

Briefly, cells transfected with sh‐KIAA1429 or treated with autophagy‐linked reagent (CQ) were collected. Cells were pre‐fixed with glutaraldehyde (2.5%) and fixed with osmium tetroxide (1%). Afterwards, as acetone and ethanol concentrations increase, the samples were gradually dehydrated and embedded in araldite. Lastly, sections (50–60 nm) were prepared on a microtome and transferred to the copper network for observation under a transmission electron microscope.

### 2.15 Measurement of autophagic flux

Cells were introduced with the GFP‐RFP‐LC3 plasmid. After treatment with autophagy‐related drug (CQ), fluorescence images of each sample were obtained using a confocal laser scanning microscope.

### 2.16 Lysotracker Red staining

3 × 10^5^ cells/well were planted and cultured in 6‐well plates. After transfection with sh‐KIAA1429 or treatment with chloroquine, cells were treated with a lysine‐tracking red probe for 15 min of incubation. Pictures were taken using a fluorescence microscope (Olympus, Toyko, Japan).

### 2.17 Statistical analysis

The survival curve of patients was tested using the Kaplan–Meier method. The relationship between clinicopathological features and KIAA1429 expression was analyzed by chi‐squared test or Fisher's test. The differences between intergroup were analyzed using one‐way ANOVA and Student's *t*‐test. Statistical analyses were conducted using SPSS software. Data are presented as means ± SD. *p* < 0.05 was statistically significant.

## 3 RESULTS

### 3.1 m^6^A methyltransferase KIAA1429 is highly expressed in lung cancer samples

**FIGURE 1:**
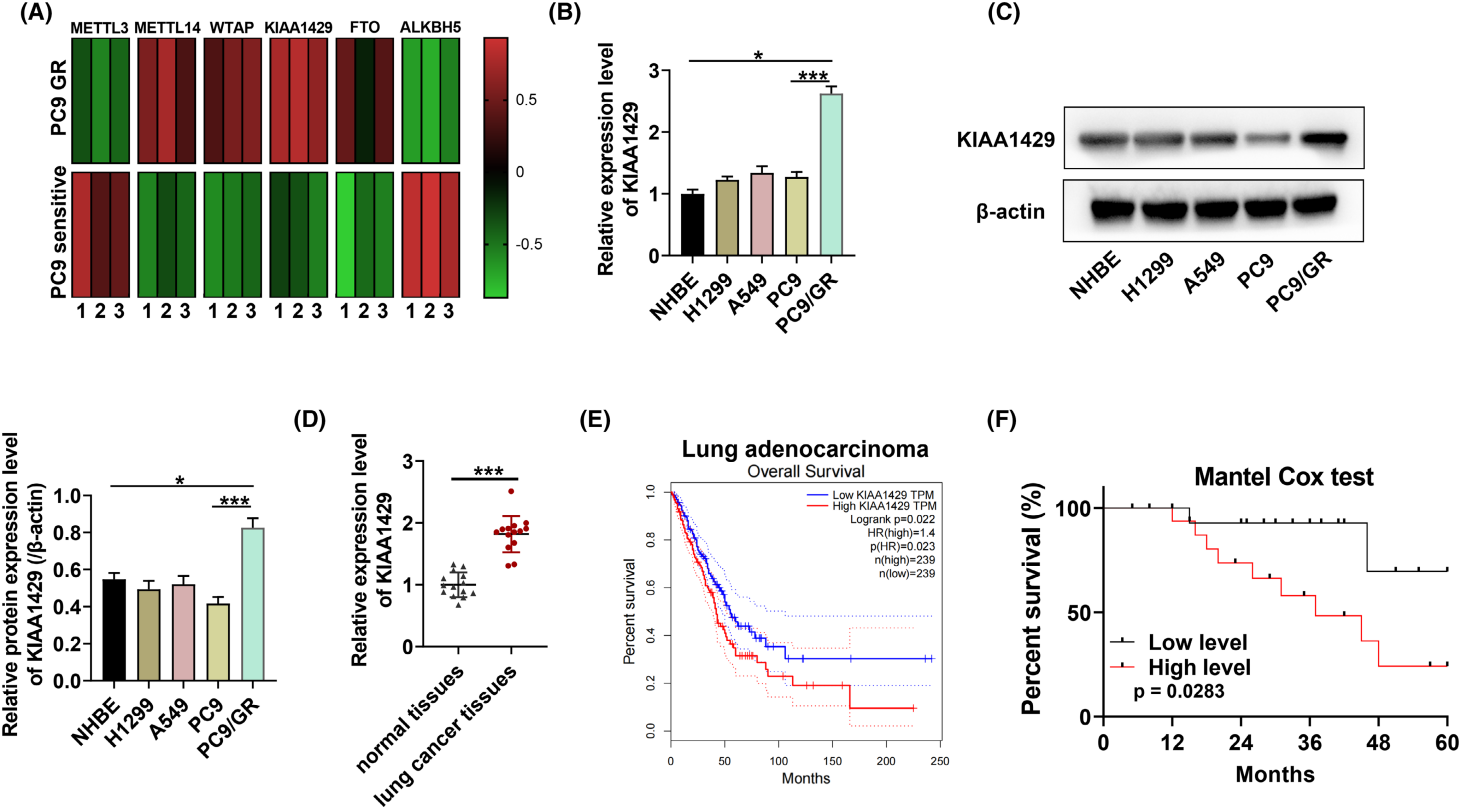
m^6^A methyltransferase KIAA1429 is highly expressed in lung cancer. (A) Expression of m6A regulators in parental sensitive cells as well as gefitinib‐resistant NSCLC cells from heatmap. (B, C) RT‐qPCR and western blot assay of KIAA1429 expression in different NSCLC cell lines and PC9‐GR. (D) KIAA1429 expression in NSCLC patients' samples along with healthy from RT‐qPCR. (E) TCGA database (http://gepia.cancer‐pku.cn/) indicated the survival of lung cancer patients with KIAA1429 expression. (F) The clinical cohort for the survival of NSCLC patients with KIAA1429 expression. *p < 0.05, ***p < 0.001.

We detected several candidate m6A modulators in PC9‐GR compared to parental cells. As revealed in Figure 1A, KIAA1429 was highly expressed in PC9‐GR cells. Besides, in comparison with NSCLC cell lines (A549, H1299, and PC9), KIAA1429 was expressed higher in PC9‐GR cells (Figure 1B). KIAA1429 protein expression was confirmed to be upregulated in PC9‐GR cells, but not significantly altered in other NSCLC cell lines (A549, H1299, and PC9) (Figure 1C). Otherwise, KIAA1429 overexpressed in NSCLC tissue samples (Figure 1D). In addition, survival rates were lower in NSCLC patients with high KIAA1429 expression (Figure 1E,F).

### 3.2 KIAA1429 promotes the proliferation as well as gefitinib resistance of NSCLC cells

**FIGURE 2:**
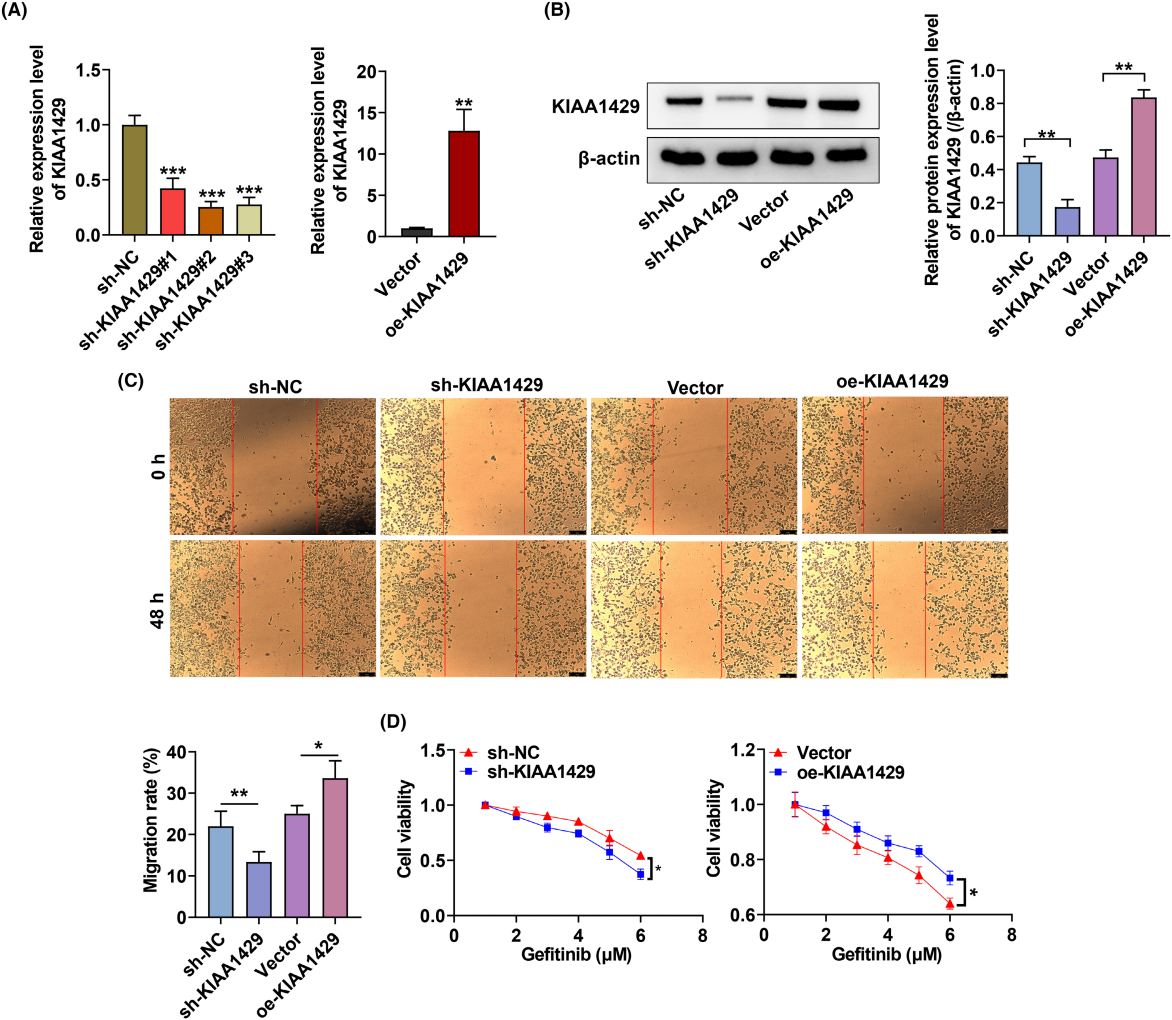
KIAA1429 knockdown suppresses the proliferation and overcomes gefitinib resistance of NSCLC cells. (A, B) Transfection efficiency of KIAA1429 in PC9‐GR cells using RT‐qPCR together with western blot. (C) Wound‐healing assay assessed PC9‐GR cells migration after KIAA1429 depletion or increase. (D) IC50 value of gefitinib concentration was assessed. *p < 0.05, **p < 0.01, ***p < 0.001.

Based on RT‐qPCR, we validated that the expression of KIAA1429 in PC9‐GR cells decreased or increased after transfection KIAA1429 shRNAs or the corresponding plasmids (Figure 2A). After transfection of the plasmid with the constructed sh‐KIAA1429 or overexpression plasmid, the KIAA1429 protein expression was confirmed to be silenced or upregulated (Figure 2B). Wound‐healing assays manifested that KIAA1429 silencing suppressed the migration properties of NSCLC cells, but KIAA1429 overexpression promoted the NSCLC cells migration (Figure 2C). We also found that KIAA1429 knockdown lessened the IC50 value of gefitinib, but KIAA1429 elevation enhanced the IC50 value (Figure 2D).

### 3.3 m^6^A is highly enriched in PC9‐GR cells

**FIGURE 3:**
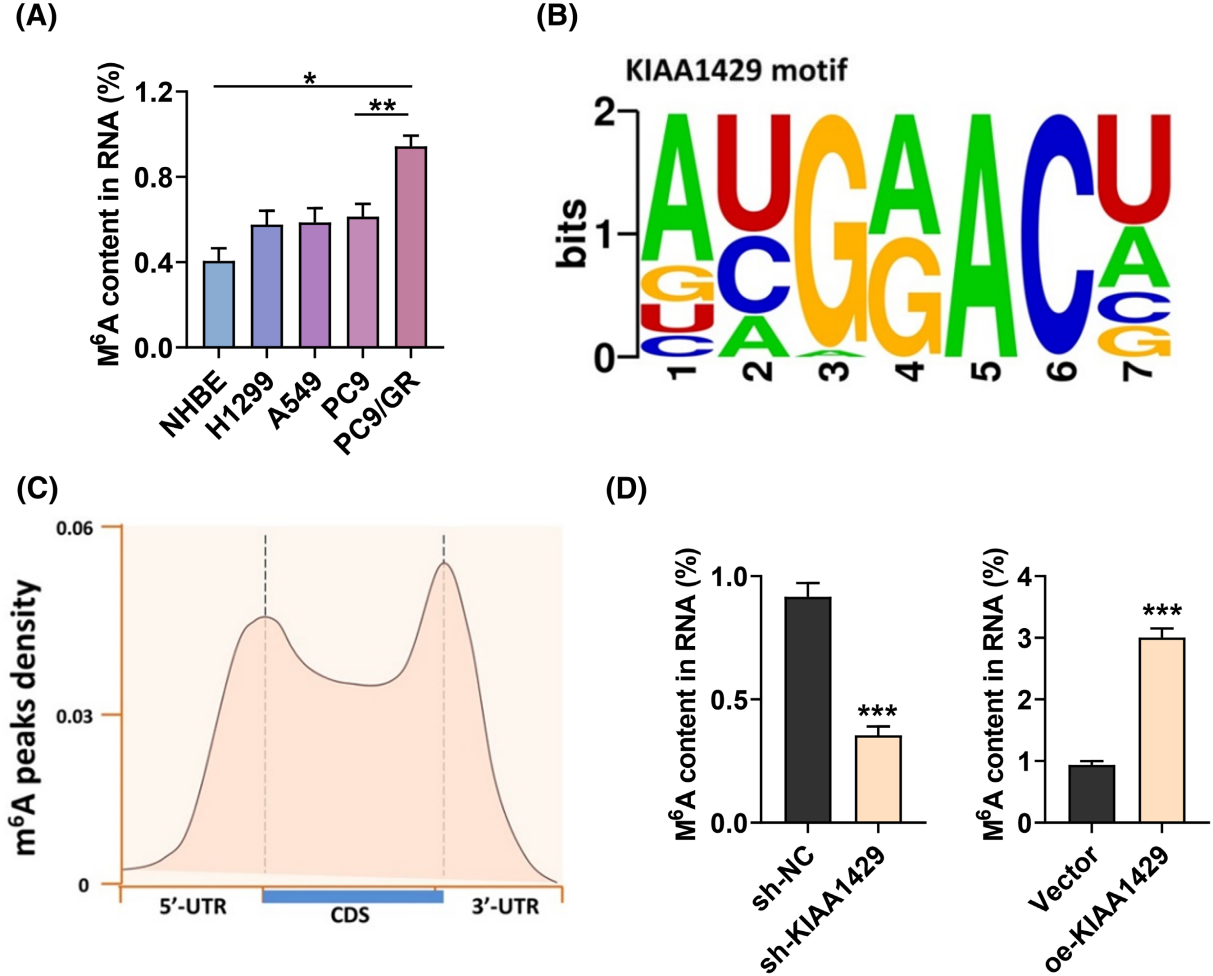
MeRIP‐Seq discloses the m6A profile in gefitinib‐resistant NSCLC cells. (A) m6A quantitative analysis was conducted to explicate m6A enrichment in PC9 and PC9‐GR cells. (B)Top sequence motif identified from MeRIP‐seq peaks in control and KIAA1429‐depleted cells. (C) MeRIP‐Seq was used to detect the metagene profile of m6A distribution. (D) Quantitative analysis of m6A in PC9‐GR cells upon KIAA1429 silence or KIAA1429 increase. *p < 0.05, **p < 0.01, ***p < 0.001.

We further explored the mechanisms KIAA1429 regulate gefitinib resistance in NSCLC cells. We quantified m6A, which was enriched in PC9‐GR cells compared to NSCLC cell lines (A549, H1299, and PC9) (Figure 3A). Subsequently, to gain insight into the regulatory implications of KIAA1429 in integrated gene expression, MeRIP‐Seq analysis was performed in NSCLC cells transfected with sh‐KIAA1429 and control sh‐NC. The “AUGGACU” sequence motif was verified to be highly enriched in m6A immunoprecipitated RNAs (Figure 3B). Since KIAA1429 actively mediates m6A modification, it is theoretically expected that the 1152 decreased peaks would include the genuine targets of KIAA1429.^25^ Therefore, we focused on mRNA transcripts with these reduced m6A peaks and found that they were predominantly distributed throughout the transcriptome (Figure 3C). Moreover, knockdown KIAA1429 suppressed m6A levels in PC9‐GR cells, but its elevation promoted m6A level (Figure 3D).

### 3.4 KIAA1429 promotes the stability of WTAP mRNA

**FIGURE 4:**
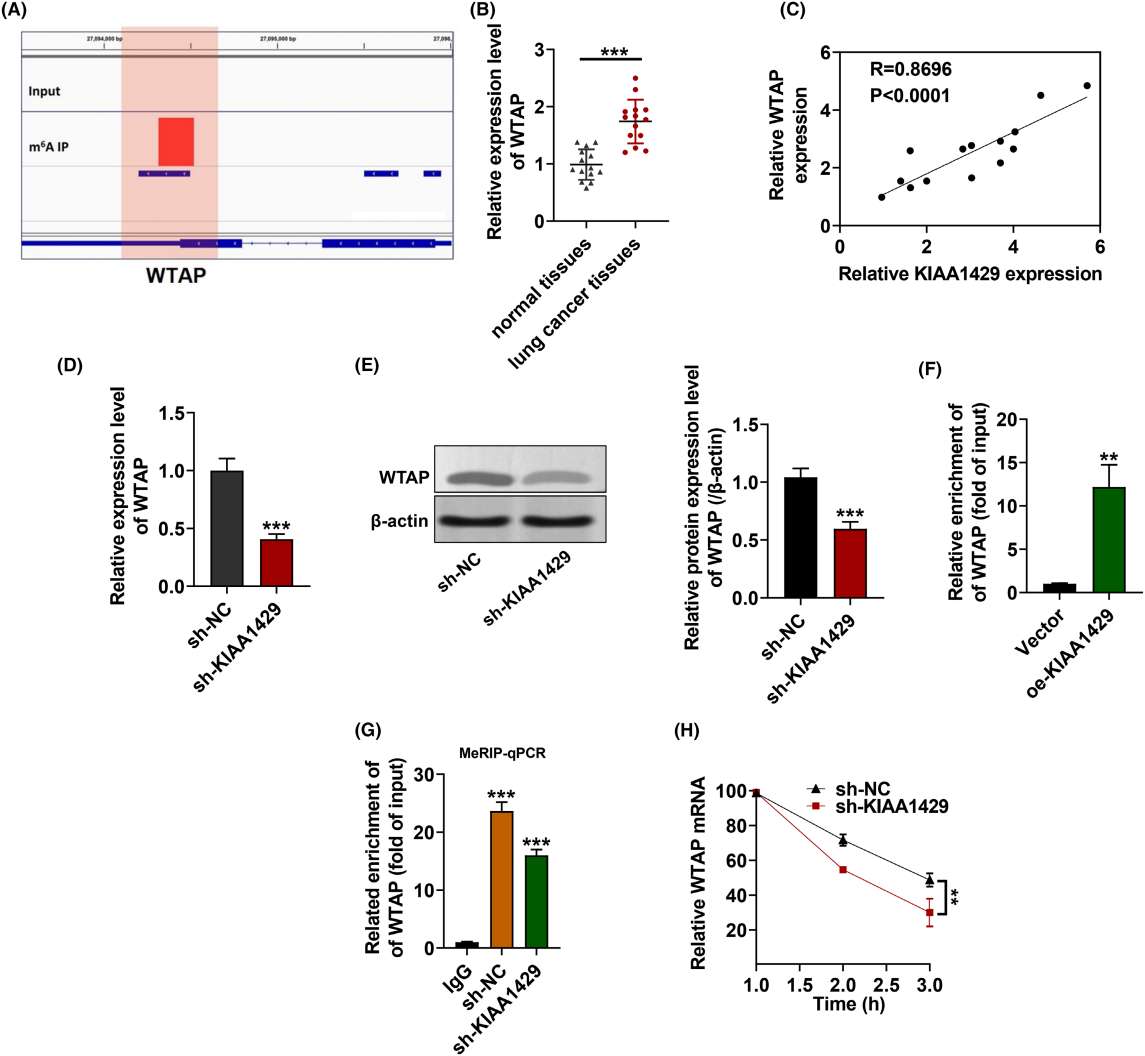
KIAA1429 stabilizes WTAP mRNA. (A) The integrative genomics viewer (IGV) tool of the marked m6A peak in WTAP mRNA 3′UTR. (B) WTAP expression in NSCLC patients' samples along with healthy from RT‐qPCR. (C) Correlation analysis of WTAP and KIAA1429 relative expression level in the 14 NSCLC tissues. (D‐E) RT‐qPCR and western blot detection of WTAP mRNA expression. (F) RIP expounded the interaction of KIAA1429 and m6A‐modified WTAP mRNA. (G) Primer‐specific MeRIP‐qPCR for WTAP mRNA revealed the m6A modification level due to KIAA1429 silence. (H) The decay rate of RNA was identified in KIAA1429‐silenced PC9‐GR cells treated with 1 μg/mL actinomycin D. using qPCR. **p < 0.01, ***p < 0.001.

WTAP is known as a potential target for KIAA1429,^21^ and our MeRIP‐Seq results have confirmed this conjecture. We utilized the integrated genome viewer (IGV) tool and found a significant m^6^A peak in the WTAP mRNA 3′‐UTR (Figure 4A). We thereby examined the expression level of WTAP in 14 pairs of NSCLC samples. Accordingly, WTAP expression in NSCLC tissues was significantly higher than in adjacent normal tissues (Figure 4B) and was considerably correlated with the expression of KIAA1429 (Figure 4C), implying a regulatory relationship of WTAP expression by KIAA1429. Subsequently, we measured the RNA and protein expression of WTAP after silencing KIAA1429 by transfection with lentivirus‐packaged shRNA. Stable KIAA1429 knockdown resulted in a statistically reduced WTAP abundance at RNA and protein levels (Figure 4D,E). Moreover, we measured the RNA expression of WTAP when overexpressed KIAA1429. The upregulated KIAA1429 resulted in statistically elevated WTAP abundance at RNA levels (Figure 4F), indicating that KIAA1429 is a direct upstream regulator of WTAP. We then demonstrated the role of KIAA1429 in WTAP m6A modification by MeRIP‐qPCR with specific primers and discovered that m6A modification levels were inhibited due to KIAA1429 reduction (Figure 4G). Interestingly, the stability of the WTAP mRNA decreased after KIAA1429 silencing (Figure 4H).

### 3.5 The KIAA1429/WTAP axis facilitates the proliferation along with gefitinib resistance of NSCLC cells

**FIGURE 5:**
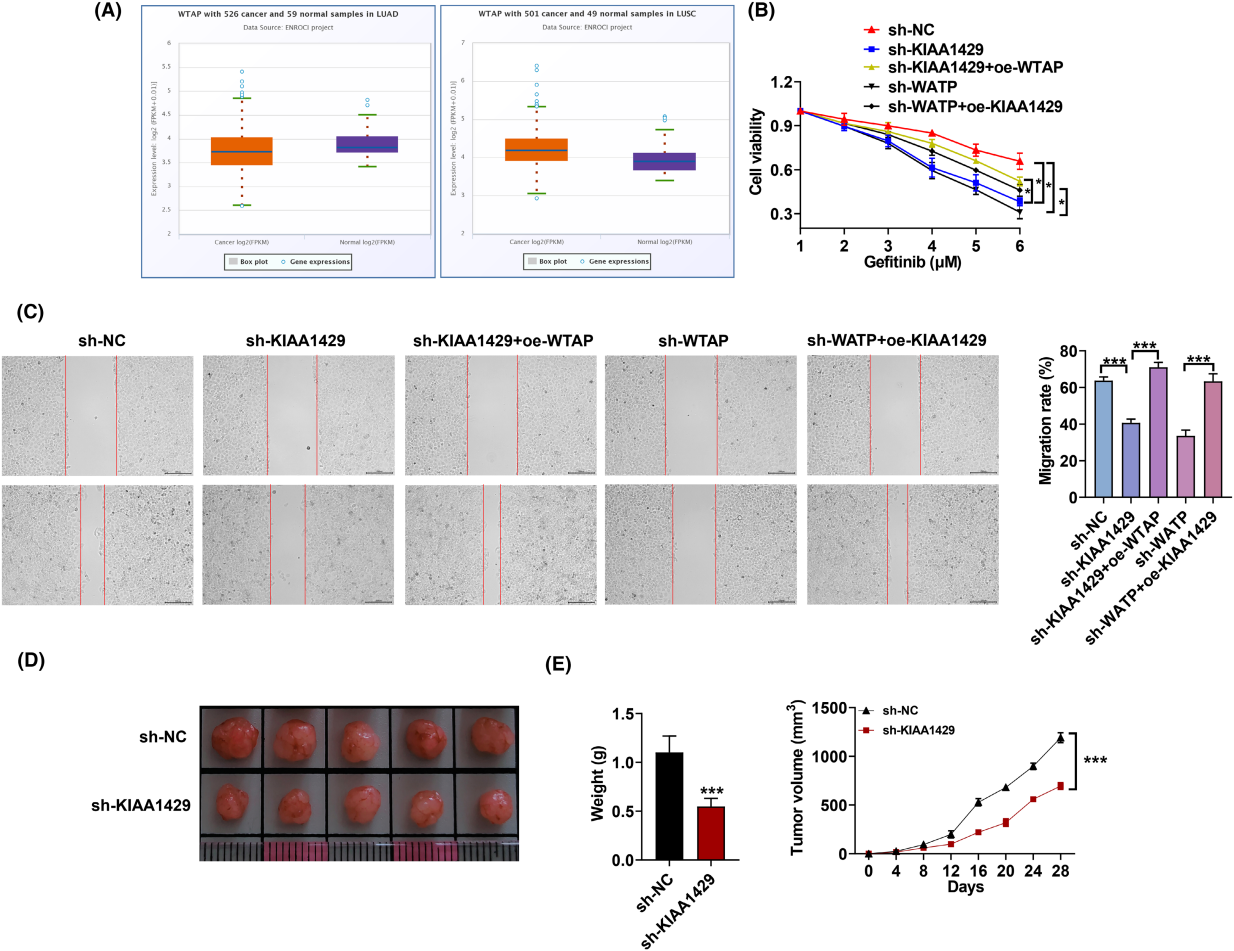
KIAA1429/WTAP axis promotes NSCLC cells proliferation as well as gefitinib resistance. (A) WTAP expression in lung cancer from TCGA database (http://gepia.cancer‐pku.cn). (B) IC50 value of gefitinib resistance in PC9‐GR cells upon KIAA1429 depletion, WTAP overexpression, si‐WTAP, and KIAA1429 OV. (C) Wound‐healing assay in PC9‐GR cells upon transfecting sh‐KIAA1429, WTAP overexpression, si‐WTAP, and KIAA1429 OV. (D) Tumor weight as well as (E) volume was assessed in mice bearing either PC9‐GR cells after KIAA1429 silence. *p < 0.05, ***p < 0.001.

Data from the ENCORI database showed that WTAP was highly expressed in lung cancer (Figure 5A). KIAA1429 or WTAP knockdown decreased the IC50 values of PC9‐GR cells after treatment with gefitinib, but this effect was rescued after co‐overexpression of KIAA1429 or WTAP (Figure 5B). Likewise, co‐treatment of WTAP or KIAA1429 overexpression could reverse the reduced migration caused by KIAA1429 or WTAP silencing (Figure 5C). In addition, KIAA1429 silencing lessened tumor weight and volume (Figure 5D,E).

### 3.6 KIAA1429 knockdown reverses gefitinib resistance in NSCLC cells through inhibiting autophagy

**FIGURE 6:**
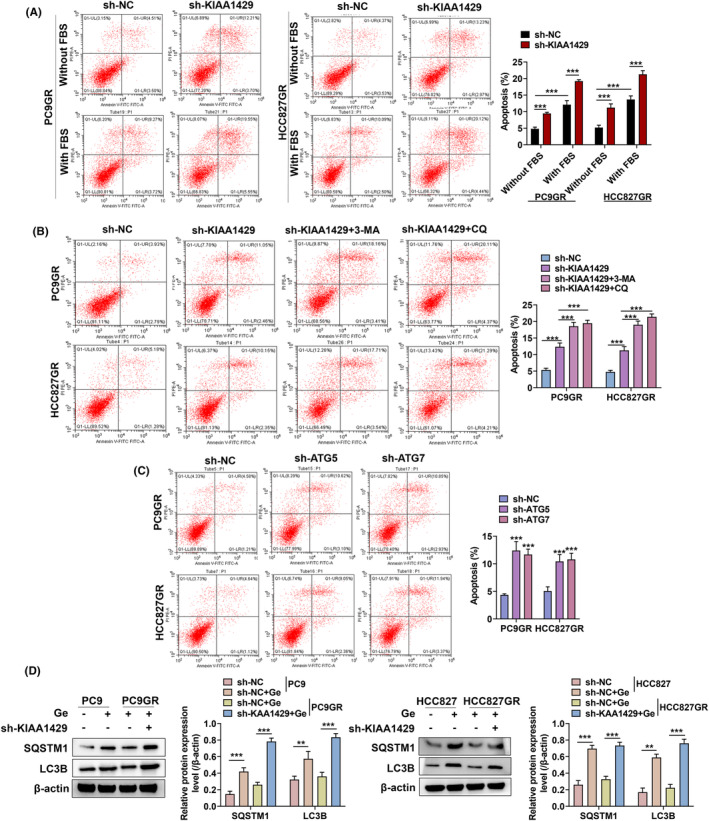
KIAA1429 knockdown reverses gefitinib resistance by lessening autophagy. (A) HCC827GR together with PC9GR cells apoptosis were determined by flow cytometry after KIAA1429 depletion. (B) Different drugs were administered to gefitinib‐resistant cells for 24 h in the presence of si‐KIAA1429, the apoptotic cells was evaluated by flow cytometry. (C) The apoptosis was measured by flow cytometry upon altering ATG5 or ATG7 expression in gefitinib‐resistant cells. (D) Western blot examined autophagy‐linked protein expression after administering Ge (gefitinib) with or without KIAA1429 knockdown. **p <0.01, ***p < 0.001.

We further cultured NSCLC cells in serum‐free medium to induce autophagy and investigate the effect of silencing KIAA1429 by flow cytometry. With or without silencing KIAA1429, cells in FBS‐free medium had fewer apoptotic cells than in FBS‐containing medium. The contrary outcomes were observed after silencing KIAA1429 (Figure 6A). These findings implied that FBS‐free media could promote autophagy, while silenced KIAA1429 could suppress autophagy. Besides, gefitinib‐resistant cells are more sensitive when KIAA1429 was silenced in medium without FBS than in FBS‐containing medium. Flow cytometry analysis showed enhanced apoptosis after silencing KIAA1429, while chloroquine or 3‐MA combined with silencing KIAA1429 resulted in a more significant increase in apoptosis (Figure 6B). The above results suggested that silencing KIAA1429 could repress the viability of gefitinib‐resistant cells by inhibiting autophagy. We downregulated the expression of ATG5 or ATG7 in gefitinib‐resistant NSCLC cells. As shown in Figure 6C, downregulated ATG5 or ATG7 elevated PC9‐GR cells apoptosis. Additionally, ATG5 or ATG7 overexpression restored the cells apoptosis. Moreover, as demonstrated in Figure 6D, SQSTM1 as well as LC3B‐II expression was elevated after silencing KIAA1429 in combination with gefitinib.

### 3.7 m^6^A methyltransferase WTAP involved in regulating NSCLC‐gefitinib resistance autophagy

**FIGURE 7:**
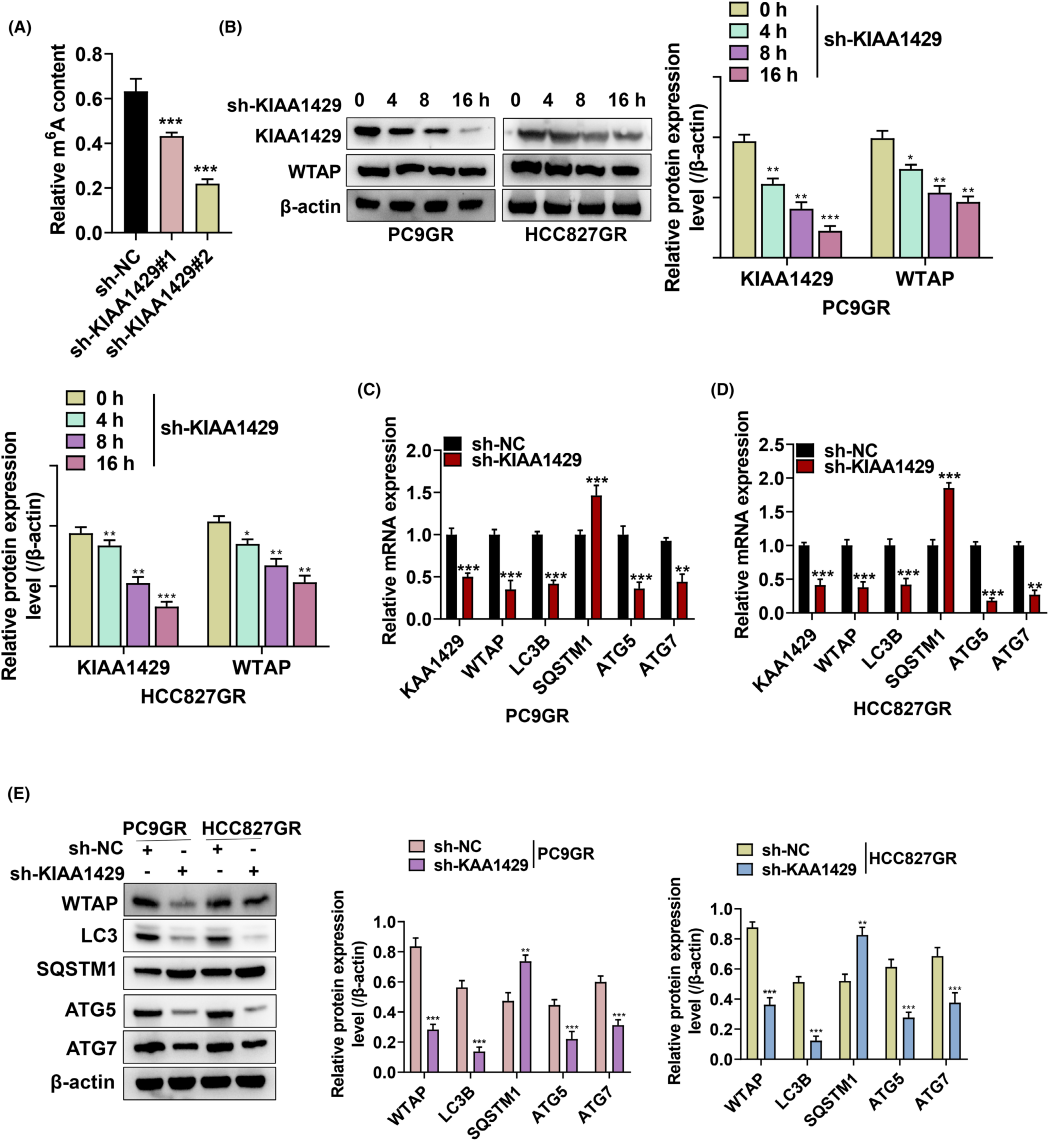
M6A methyltransferase KIAA1429 is implicated in modulating cell autophagy. (A) m6A methylation level was detected in PC9GR cells transfected with gefitinib upon KIAA1429 silence. (B) Western blot analysis of KIAA1429 together with WTAP expression due to silencing KIAA1429. (C–E) RT‐qPCR and western blot of autophagy‐related mRNA expression after silenced KIAA1429. *p <0.05, **p < 0.01, ***p < 0.001.

We investigated the role of WTAP in KIAA1429‐regulated autophagy. As shown in Figure 7A, silencing KIAA1429 suppressed m6A methylation levels in PC9‐GR cells. Since m6A methyltransferase is well‐known to install m6A to target RNAs, we speculated that silencing KIAA1429 reversed gefitinib resistance by modulating WTAP. The results showed that silencing KIAA1429 significantly inhibited the expression of WTAP (Figure 7B). Next, we discovered that the depletion of KIAA1429 decreased WTAP, LC3B, ATG5, as well as ATG7 expression but increased the expression of SQSTM1 (Figure 7C–E).

### 3.8 KIAA1429 knockdown reduces autophagy flux in NSCLC cells

**FIGURE 8:**
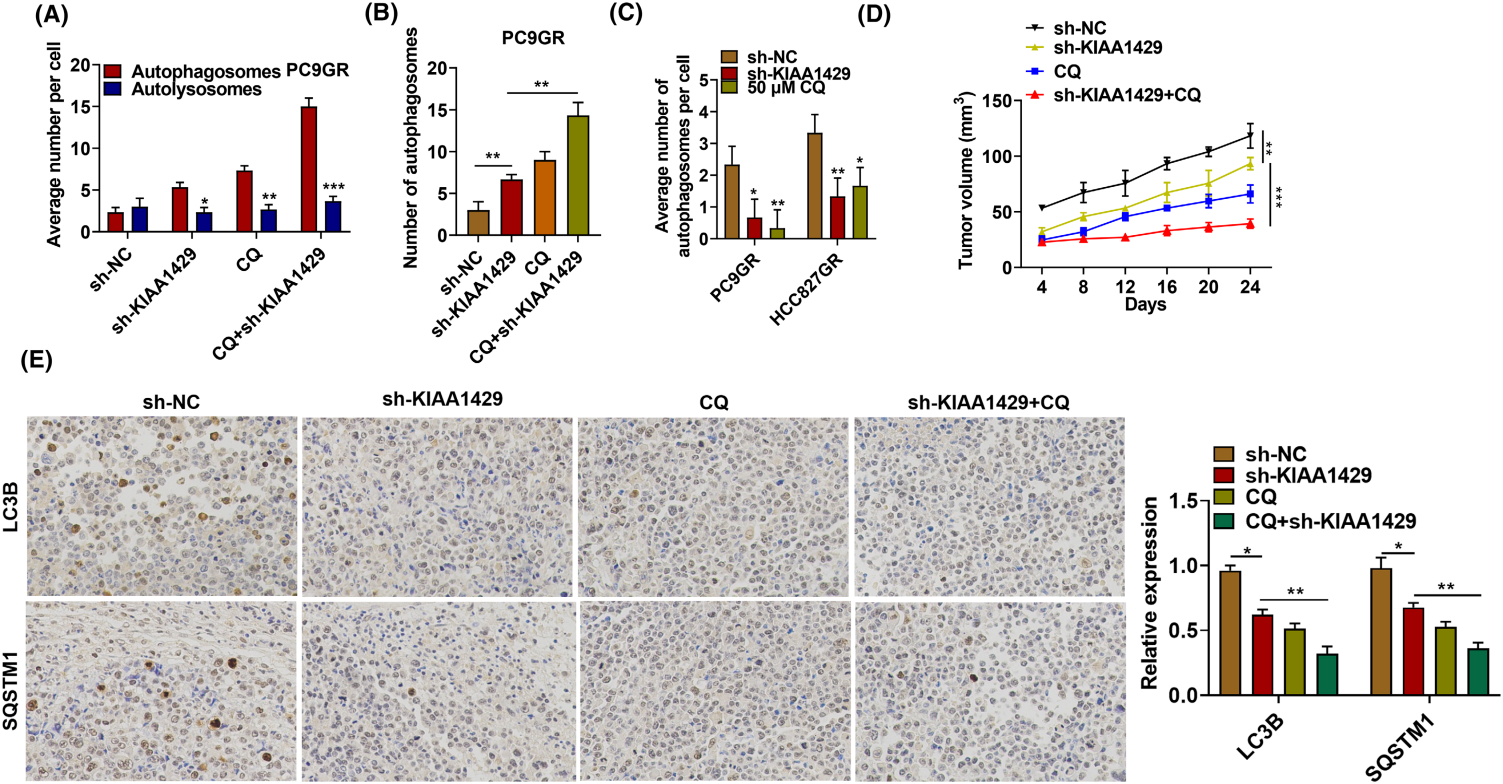
KIAA1429 knockdown blocks autophagy in NSCLC cells. (A) Immunofluorescence detected NSCLC cells treated with GFP‐RFP‐LC3 as well the indicated autophagy‐related drugs. (B) PC9GR cells after KIAA1429 silence treated with chloroquine were examined for cellular autophagic indicators by transmission electron microscopy. (C) PC9GR together with HCC827GR cells were silenced against KIAA1429 or treated with chloroquine, stained with lysotracker red dye, followed by fluorescence microscopy. (D) Effect of silencing KIAA1429, chloroquine treatment, or silencing KIAA1429 together with chloroquine treatment on tumor volume. (E) Immunohistochemical detection of LC3B as well as SQSTM1 expression in tumor tissues after silencing KIAA1429, chloroquine treatment, or silencing KIAA1429 and chloroquine treatment. *p < 0.05, **p < 0.01, ***p < 0.001.

We further investigated the mechanism by which KIAA1429 regulated autophagy by transfecting NSCLC cells with the GFP‐RFP‐LC3 plasmid. As shown in Figure 8A, KIAA1429 knockdown could increase autophagosomes, but not autolysosomes, comparable to chloroquine, a classical inhibitor of autophagy flux. Using transmission electron microscopy, KIAA1429 role in autophagy was determined by detecting the formation of autophagic vacuoles after silencing KIAA1429 or treatment with chloroquine. As shown in Figure 8B,C, silencing KIAA1429 resulted in the inhibition of autolysosome formation. Silencing KIAA1429 could suppress autophagy via attenuating lysosomal acidification. Otherwise, nude mice model harboring PC9GR cells was constructed and discovered to show that the mutes harvested showed a significantly reduction in mean tumor volume in combination chloroquine treatment KIAA1429 control or drug‐treated groups (Figure 8D). We investigated the expression of SQSTM1 and LC3B by immunohistochemistry to analyze autophagic flux in low levels KIAA1429 tumors. As shown in Figure 8E, tumors expressing low KIAA1429 have accordingly reduced expression of LC3B and SQSTM1, indicating that autophagy flux in tumors is blocked.

## 4 DISCUSSION

Emerging literature suggested that dysregulated epigenetic modifications play a crucial role in human cancers.^26^, ^27^ RNA modification, particularly m6A modifications, has been documented to be associated with drug resistance in NSCLC.^28^, ^29^ The present investigation focused on the regulatory mechanisms of m6A methyltransferase KIAA1429 in gefitinib resistance in NSCLC.

Although the antigenic or oncogenic modulations of m6A important regulators in cancers have been elucidated, the potential for deep‐insights into other pathophysiology remains bewildering.^30^, ^31^, ^32^ M6A modification in RNA has been suggested to be involved in epigenetic regulation. METTL3 positively regulates JUNB mRNA stability as well as m6A modification enrichment to affect epithelial‐mesenchymal transition in lung cancer cells.^33^ Besides, the m6A demethylase ALKBH5 quells the m6A modification abundance of FOXM1 mRNA to elevate FOXM1 expression.^34^ All above findings conclude that m6A regulators can significantly regulate tumorigenesis.

Our results showed that m6A methyltransferase KIAA1429 upregulated in PC9‐GR cells. High expression of KIAA1429 represented unfavorable outcome in patients with NSCLC. Besides, KIAA1429 promoted PC9‐GR cell migration. Mechanistically, silencing KIAA1429 elevates the IC50 value of PC9‐GR cells after treatment with gefitinib. These results proved that KIAA1429 may modulate the resistance of NSCLC cells to gefitinib.

Numerous analogous evidence certified the function of m6A in human chemoresistant tumors. For example, WTAP promotes autophagy and inhibits hepatocellular carcinoma cell proliferation^22^ and is a target gene for KIAA1429.^21^ Many reports have verified that autophagy activation facilitates anti‐cancer drug resistance to increase cell survival, while suppressed autophagy elevates the sensitivity of cancer cells to anti‐cancer drugs.^35^ For instance, chidamide declines c‐MET expression by reducing mRNA m6A methylation modification via downregulating METTL3 and WTAP expression.^36^ We found that KIAA1429 interacted with WTAP, whose silencing suppressed WTAP expression and promoted autophagy in gefitinib‐resistant NSCLC in vitro and in vivo.

In the current work, KIAA1429 could target the WTAP mRNA 3′‐UTR. Downregulated KIAA1429 results in abnormal RNA metabolism in oocytes.^37^ In gastric cancer, KIAA1429 accelerates proliferation via stabilizing c‐Jun mRNA through a m6A‐independent way.^38^ Therefore, the modulation of KIAA1429 in gefitinib‐resistant NSCLC is highly credible.

Conclusively, the present investigation discovered that KIAA1429 was highly expressed in gefitinib‐resistant NSCLC cells and was related to adverse clinical outcomes. KIAA1429 enhanced the resistance of NSCLC cells to gefitinib and elevated the mRNA stability of WTAP. Silencing of KIAA1429 suppressed the expression of WTAP and promoted autophagy. Overall, these findings convincingly provided the KIAA1429/WTAP axis as a feasible target for treatment of NSCLC patients.

## AUTHOR CONTRIBUTIONS

Bo Ma and Lili Ding made majority contribution to the conception of this study, Lei Xiu and Bo Ma carried out all of experiments, Bo Ma prepared the first draft of this manuscript. Lili Ding agreed the final design of this work and revised this manuscript critically. All authors have read and approved the final manuscript.

## FUNDING INFORMATION

No funding.

## CONFLICT OF INTEREST STATEMENT

The authors declare no conflicts of interest.

## ETHICS STATEMENT

The experiments were reviewed and approved by the Ethics Committee of General Hospital of Ningxia Medical University.

## Data Availability

The datasets used and/or analyzed during the current study are available from the corresponding author on reasonable request.
